# Cerebroplacental Ratio Versus Nonstress Test in Predicting Adverse Perinatal Outcomes in Hypertensive Disorders of Pregnancy: A Prospective Observational Study

**DOI:** 10.7759/cureus.26462

**Published:** 2022-06-30

**Authors:** Priyadarshini Nayak, Sweta Singh, Pruthwiraj Sethi, Tapas Kumar Som

**Affiliations:** 1 Obstetrics and Gynecology, All India Institute of Medical Sciences, Bhubaneswar, IND; 2 Neonatology, All India Institute of Medical Sciences, Bhubaneswar, IND

**Keywords:** specificity, sensitivity, hypertensive disorders of pregnancy, cerebroplacental ratio, nonstress test

## Abstract

Introduction: In developing countries, nonstress test (NST) is the most widely used method for antenatal fetal surveillance.Lately, cerebroplacental ratio (CPR) has emerged as a predictor for adverse perinatal outcomes, especially in hypertensive disorders in pregnancy (HDP). Against this background, the present study was conducted with the primary objective of quantifying the diagnostic accuracy of cerebroplacental ratio (CPR) versus nonstress test (NST) in predicting adverse perinatal outcomes in women with HDP.

Methods: This was a prospective observational cohort study conducted in a tertiary care institute in eastern India. All consecutive women with hypertension in pregnancy at a gestational age of ≥32 weeks were recruited into the study. Both CPR and NST were performed at baseline and repeated weekly till delivery. The parameters obtained within one week of delivery were entered for analysis.

Results: Sixty-two of the 65 women completed the study. There were 22 women (35.5%) in group A (both CPR and NST normal), 17 (27.4%) in group B (CPR abnormal, NST normal), 14 (22.6%) in group C (CPR normal and NST abnormal), and nine (14.5%) in group D (both CPR and NST abnormal). CPR had greater sensitivity (93.33% versus 46.67%), with higher positive predictive value (53.85% versus 30.43%), specificity (74.47% versus 65.91%), and negative predictive value (97.22% versus 79.49%) than NST for predicting neonatal intensive care unit admission. CPR also had higher sensitivity (84.62% versus 61.54%) and specificity (91.34% versus 69.39%) than NST in predicting neonatal complications. The negative predictive value (NPV) of CPR was 100% for predicting requirement of bag and mask ventilation and continuous positive airway pressure.

Conclusion: CPR had greater diagnostic accuracy in terms of both higher sensitivity and greater specificity than NST in predicting adverse perinatal outcomes in women with hypertensive disorders of pregnancy.

## Introduction

Hypertensive disorders of pregnancy (HDP) complicate around 10% of all gestations in sub-Saharan Africa and Southern Asia, with gestational hypertension accounting for 6-8%, preeclampsia 2-4%, chronic hypertension 1-3%, and eclampsia less than 1% of the pregnancies [1]. HDP contributes significantly to maternal and perinatal morbidity and mortality across the globe [1,2].

In developing countries, nonstress test (NST) is the most widely used method for antenatal fetal assessment and for predicting adverse perinatal outcomes in high-risk pregnancies due to its ready availability, ease of use, requirement of minimal time, and low costs [3,4]. Of late, the cerebroplacental ratio (CPR) has emerged as a predictor for adverse perinatal outcomes, especially in HDP [5,6]. Previous studies have compared NST versus CPR in either preeclampsia, severe preeclampsia, or gestational hypertension alone [7-10]. Literature comparing both NST and CPR for predicting adverse perinatal outcomes for the entire spectrum of HDP is scarce.

Thus, the present study was carried out with the primary objective of quantifying the diagnostic accuracy of CPR versus NST in predicting adverse perinatal outcomes in women with HDP.

## Materials and methods

Study design and settings

This prospective observational cohort study was conducted in the department of obstetrics and gynecology of a tertiary teaching hospital in eastern India. Ethical approval was obtained prior to the commencement of the study (Institutional Ethics Committee, All India Institute of Medical Sciences, Bhubaneswar, Reference number: IEC/AIIMS BBSR/PG Thesis/2017-18/27).

Inclusion and exclusion criteria

All consecutive antenatal hypertensive women at a gestational age of ≥32 weeks were recruited into the study. Hypertension in pregnancy was defined as a blood pressure reading of ≥140/90 mmHg, on two occasions, few hours apart, and was classified as per the International Society for the Study of Hypertension in Pregnancy (ISSHP) classification [11].

Pregnant women with diabetes, thyroid disorders, severe anemia, congenitally malformed fetus and fetal chromosomal anomaly, and small for gestational age (SGA) fetus or fetal growth restriction (FGR) not due to HDP were excluded from the study.

Assuming the prevalence of HDP as 10%, the probability of obtaining an abnormal CPR in women with HDP as 40% and that of abnormal nonstress test also as 40%, diagnostic odds ratio of six, confidence level of 95%, and power of 80%, the sample size obtained was 58. Considering a nonresponse rate of 10%, the sample size was calculated as 65.

Study protocol

After recruitment into the study, a detailed history was obtained and clinical examination, along with relevant investigations was performed. The maternal parameters recorded were age, obstetric score, gestational age, systolic blood pressure, diastolic blood pressure, proteinuria, hemoglobin, total platelet count, aspartate transaminase, alanine transaminase, creatinine, uric acid, and fundoscopy. Based on this, women were classified as having chronic hypertension, preeclampsia, eclampsia, and gestational hypertension.

At recruitment, a baseline obstetric ultrasound with Doppler as well as NST was performed for each participant. Obstetric ultrasound was performed using the Mindray ultrasound equipment (Shenzhen, China: Mindray Bio-Medical Electronics Co., Ltd.), models no. 365A and UMT-150, in the outpatient department and labor ward, respectively, with a 3.5 MHz convex transducer, with the woman in semi-recumbent position and 15 degrees left lateral tilt by the investigators. Doppler transducer was placed on the maternal abdomen over the uterus and manipulated till Doppler signals appropriate for the middle cerebral artery (MCA) and umbilical artery (UA) were obtained, and measured as per the International Society of Ultrasound in Obstetrics and Gynecology (ISUOG) practice guidelines [12]. CPR was then calculated as the ratio of pulsatility index (PI) of the MCA to that of the UA, with PI being defined as the peak systolic velocity - end-diastolic velocity/timed average maximum velocity, obtained in auto mode. For the purpose of this study, a value of >1 after 32 weeks gestation was taken as normal, and a value of <1 was taken as abnormal.

NST was performed by cardiotocography equipment Sonicaid Team Duo in the labor ward. For performing the NST, the fetal heart rate transducer was belted on the maternal lower abdomen at a site where the fetal heart rate was most distinctly audible and toco transducer was belted on the maternal upper abdomen over the uterine fundus with the woman in supine position with 15 degrees left lateral tilt. The NST was categorized as normal, suspicious, or pathological according to National Institutes of Clinical Excellence (NICE) clinical guidelines [3]. For the purpose of this study, a pathological NST was taken as abnormal.

The CPR and NST were repeated for each participant at weekly intervals, with the pregnancy planned for termination at 37 completed weeks of gestation, unless the maternal or fetal condition necessitated earlier delivery. Magnesium sulfate was used as an anticonvulsant in severe preeclampsia and eclampsia, and not for fetal neuroprotection, as our gestational age at recruitment was ≥32 weeks. Antenatal corticosteroids were used till 34 weeks only. Obstetric management was done as per standard institutional protocol.

The parameters obtained within one week of delivery were entered for analysis. The recruited women were divided into four groups based on the last NST and CPR as follows - group A: both CPR and NST were normal, group B: CPR was abnormal but NST was normal, group C: CPR was normal but NST was abnormal, and group D: both CPR and NST were abnormal.

The perinatal outcomes recorded were delivery by lower segment cesarean section or vaginal delivery, baby weight, low or normal birth weight, Apgar scores at 1 and 5 minutes, admission to neonatal intensive care unit (NICU), neonatal complications like hypoglycemia, polycythemia, neonatal asphyxia, acidosis, neonatal sepsis, requirement of bag and mask ventilation (BMV), requirement of continuous positive airway pressure (CPAP), neonatal deaths, and the total duration of hospital stay.

Statistical analysis

Data were entered in MS Excel spreadsheets (Microsoft office 2016). SPSS version 22 (Armonk, NY: IBM Corp.) was used for data analysis. Descriptive analysis of categorical variables was presented as frequency with percentage (%). Continuous variables were expressed as mean with standard deviation. Chi-squared test was used for group comparisons of categorical data. In case, the expected frequency in the contingency tables was found to be <5 for >25% of the cells, Fisher’s exact test was used instead. Group comparison for continuous variables was done using the Kruskal-Wallis test. The diagnostic accuracy of both the tests was compared by estimating sensitivity, specificity, positive predictive value (PPV), and negative predictive value (NPV). Statistical significance was kept at p<0.05.

## Results

**Figure 1:**
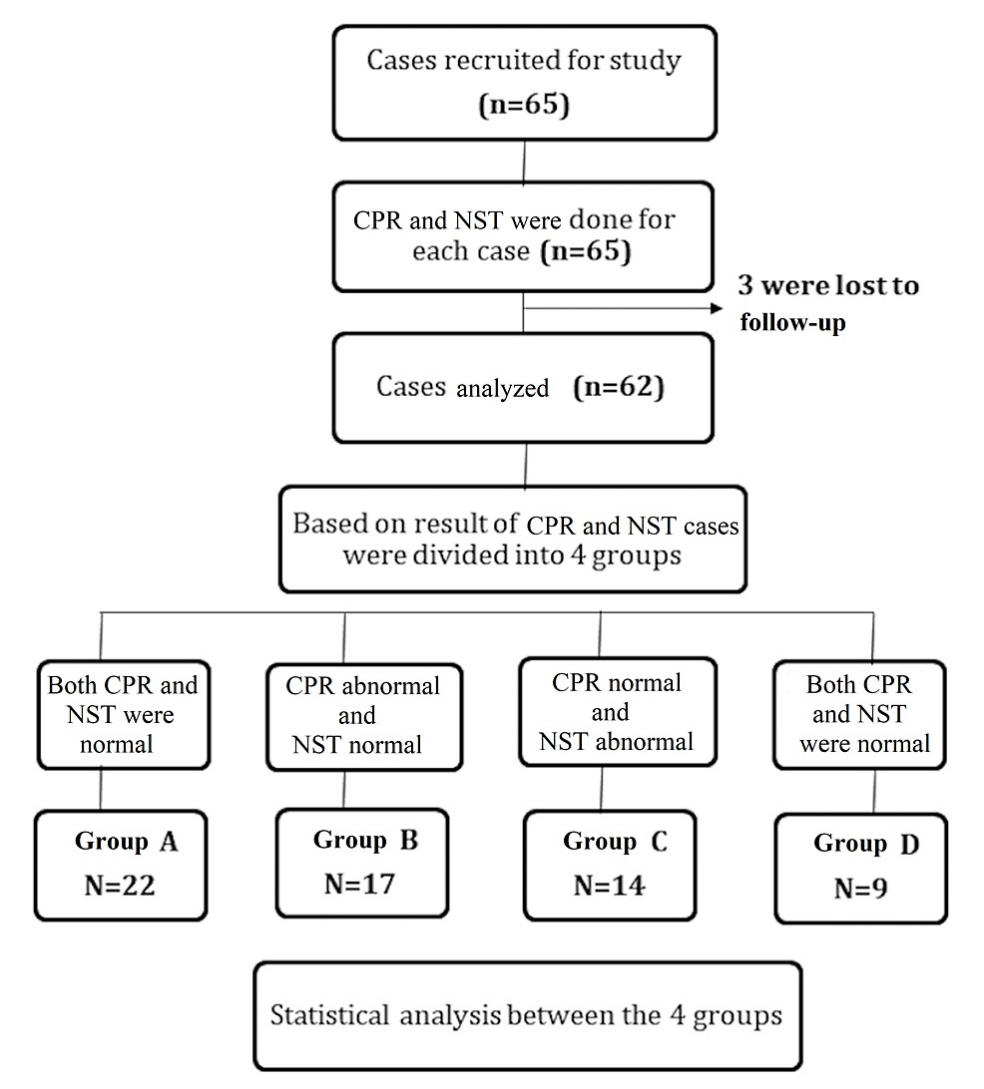
Strengthening the reporting of observational studies in epidemiology (STROBE) flow diagram. NST: nonstress test; CPR: cerebroplacental ratio

Sixty-five women with HDP at ≥32 weeks of gestation were included in the study over a period of two years. Three were lost to follow-up and excluded. Thus, 62 women completed the study (Figure 1). There were 22 women (35.5%) in group A, 17 (27.4%) in group B, 14 (22.6%) in group C, and nine (14.5%) in group D.

The groups were similar in their characteristics except for mean maternal age which was significantly higher in group D (30.56 years) and in the proportion of women delivering preterm (gestational age <37 completed weeks at delivery) which was highest in group B (70.6%). Maternal baseline characteristics are depicted in Table 1. Table 2 depicts the perinatal outcomes across the groups.

**Table 1:** Baseline maternal characteristics of the study population. *Significant at p<0.05. **Kruskal-Wallis test. ***Fisher's exact test. ****Chi-squared test. Obs: obstetric; GA: gestational age; HTN: hypertension; NS: not severe; S: severe

All parameters		Group				p-Value
		A (n=22)	B (n=17)	C (n=14)	D (n=9)	
Age*		25.64±4.32	30.35±4.91	29.93±4.16	30.56±5.05	0.005^**^
Age group	18-20	3 (13.6%)	0 (0.0%)	0 (0.0%)	1 (11.1%)	0.072^***^
	21-30	16 (72.7%)	10 (58.8%)	8 (57.1%)	3 (33.3%)	
	31-40	3 (13.6%)	7 (41.2%)	6 (42.9%)	5 (55.6%)	
Obs score	Multigravida	8 (36.4%)	10 (58.8%)	8 (57.1%)	5 (55.6%)	0.483^***^
	Primigravida	14 (63.6%)	7 (41.2%)	6 (42.9%)	4 (44.4%)	
GA*	Preterm	7 (31.8%)	12 (70.6%)	3 (21.4%)	5 (55.6%)	0.022^****^
	Term	15 (68.2%)	5 (29.4%)	11 (78.6%)	4 (44.4%)	
Diagnosis	Chronic HTN	3 (13.6%)	5 (29.4%)	1 (7.1%)	0 (0.0%)	0.155^***^
	Eclampsia	1 (4.5%)	0 (0.0%)	0 (0.0%)	0 (0.0%)	
	Gestational HTN	14 (63.6%)	5 (29.4%)	9 (64.3%)	4 (44.4%)	
	Preeclampsia	4 (18.2%)	7 (41.2%)	4 (28.6%)	5 (55.6%)	
Severity	NS	16 (72.7%)	13 (76.5%)	8 (57.1%)	4 (44.4%)	0.315^***^
	S	6 (27.3%)	4 (23.5%)	6 (42.9%)	5 (55.6%)	

**Table 2:** Perinatal outcomes of the study population. *Significant at p<0.05. **Kruskal-Wallis test. ***Fisher’s exact test. ****Chi-squared test. NST: nonstress test; UA: umbilical artery; PI: pulsatility index; MCA: middle cerebral artery; CPR: cerebroplacental ratio; LSCS: lower segment cesarean section; VD: vaginal delivery; AFI: amniotic fluid index; LBW: low birth weight; NICU: neonatal intensive care unit; BMV: bag and mask ventilation; CPAP: continuous positive airway pressure

All parameters		Group				p-Value
		A (n=22)	B (n=17)	C (n=14)	D (n=9)	
NST*	Normal	22 (100.0%)	17 (100.0%)	0 (0.0%)	0 (0.0%)	<0.001^***^
	Pathological	0 (0.0%)	0 (0.0%)	7 (50.0%)	6 (66.7%)	
	Suspicious	0 (0.0%)	0 (0.0%)	7 (50.0%)	3 (33.3%)	
UA PI*		0.89±0.15	1.62±0.70	0.95±0.21	1.35±0.41	<0.001^**^
MCA PI		1.34±0.17	1.19±0.39	1.29±0.27	1.16±0.38	0.086^**^
CPR*	<1	0 (0.0%)	17 (100.0%)	0 (0.0%)	9 (100.0%)	<0.001^****^
	>1	22 (100.0%)	0 (0.0%)	14 (100.0%)	0 (0.0%)	
LSCS/VD	LSCS	16 (72.7%)	16 (94.1%)	13 (92.9%)	8 (88.9%)	0.265^***^
	VD	6 (27.3%)	1 (5.9%)	1 (7.1%)	1 (11.1%)	
AFI*		12.30±2.88	10.86±3.11	9.08±2.86	7.07±3.87	0.001^**^
Baby weight (g)*		2720.32±544.91	2041.24±688.75	2695.43±687.16	1468.00±658.35	<0.001^**^
Baby weight	LBW	10 (45.5%)	12 (70.6%)	9 (64.3%)	8 (88.9%)	0.116^****^
	Normal	12 (54.5%)	5 (29.4%)	5 (35.7%)	1 (11.1%)	
Apgar (1')*		8.50±0.74	7.76±1.64	7.57±0.65	6.22±2.11	0.001^**^
Apgar (5')*		9.27±0.46	8.82±1.19	8.86±0.36	8.00±1.50	0.040^**^
NICU admission (yes)*		0 (0.0%)	8 (47.1%)	3 (21.4%)	6 (66.7%)	<0.001^***^
BMV (yes)*		0 (0.0%)	2 (11.8%)	0 (0.0%)	5 (55.6%)	<0.001^***^
CPAP (yes)*		0 (0.0%)	4 (23.5%)	0 (0.0%)	4 (44.4%)	<0.001^***^
Neonatal complications (yes)*		0 (0.0%)	5 (29.4%)	2 (14.3%)	6 (66.7%)	<0.001^***^
Duration of hospital stay (days)*		4.09±0.87	10.35±11.75	5.00±2.48	26.67±37.04	0.004^**^
Outcome*	Death	0 (0.0%)	0 (0.0%)	0 (0.0%)	3 (33.3%)	0.002^***^
	Discharged	22 (100.0%)	17 (100.0%)	14 (100.0%)	6 (66.7%)	

Overall, 53 (85.5%) participants had cesarean delivery, with the rates of cesarean delivery being similar across the groups. There were 39 (62.9%) neonates with low birth weight (LBW), with the highest number of LBW babies in group D (88.9%), and lowest in group A (45.5%). This represented significant difference. The mean birth weight was highest in group A (2720.32 g) and lowest in group D (1468 g), which was also statistically significant.

There was significant difference between the means of Apgar scores at 1’ and 5’ between groups A, B, and C with D and between B and C. The NICU admission rates were highest in group D (66.7%), followed by group B (47.1%), and none in group A. Similarly, the rates of BMV and requirement of CPAP were highest in group D (55.6% and 44.45, respectively), followed by group B (23.5% and 29.4%, respectively), and none in group A. Neonatal complications studied were present in 21% of the study participants in total, with the rates being highest in group D (66.7%), and none in group A. The duration of hospital stay was highest in group D (26.67 days) followed by group B (10.35 days). There were three neonatal deaths, all from group D. Table 3 depicts the comparison between the diagnostic methods of CPR and NST.

**Table 3:** Comparison of CPR with NST in the study population. CPR: cerebroplacental ratio; NST: nonstress test; PPV: positive predictive value; NPV: negative predictive value; NICU: neonatal intensive care unit; BMV: bag and mask ventilation; CPAP: continuous positive airway pressure; CI: confidence interval

Serial no.	Parameter	Diagnostic method	Sensitivity (%)	Specificity (%)	PPV (%)	NPV (%)
1	NICU admission	CPR (95% CI)	93.33 (70.18-98.81)	74.47 (60.49-84.75)	53.85 (35.46-71.24)	97.22 (85.83-99.51)
		NST (95% CI)	46.67 (24.11-69.88)	65.91 (51.67-77.83)	30.43 (15.62-50.87)	79.49 (64.47-89.22)
2	Neonatal complications	CPR (95% CI)	84.62 (57.76-95.67)	91.34 (78.26-96.24)	42.31 (25.54-61.05)	94.44 (81.86-98.46)
		NST (95% CI)	61.54 (35.52-82.29)	69.39 (55.47-80.48)	34.78 (18.81-55.11)	87.18 (73.29-94.45)
3	BMV	CPR (95% CI)	100 (64.57-100)	65.45 (52.25-76.64)	26.92 (13.72-46.08)	100 (90.36-100)
		NST (95% CI)	71.43 (35.89-91.78)	67.27 (54.13-78.19)	21.74 (96.64-41.92)	94.87 (83.11-98.58)
4	Requirement of CPAP	CPR (95% CI)	100 (67.56-100)	66.67 (53.36-77.76)	30.77 (16.52-49.99)	100 (90.36-100)
		NST (95% CI)	50 (21.52-78.48)	64.81 (51.48-76.18)	17.39 (69.79-37.14)	89.74 (76.42-95.94)

CPR was more sensitive (93.33% versus 46.67%) with higher PPV (53.85% versus 30.43%) and higher negative predictive value (97.22% versus 79.49%) than NST in predicting NICU admission. CPR also had higher sensitivity (84.62% versus 61.54%) than NST in prediction of neonatal complications. The NPV of CPR in predicting the need for BMV and CPAP was 100%.

## Discussion

This prospective study was conducted on 65 women with hypertension in pregnancy, of which 62 completed the study. CPR was found to be a good predictor of adverse neonatal outcomes than the standard NST in our study population.

In terms of baseline maternal characteristics, the mean maternal age was significantly higher in group D (30.56 years), which may represent an independent association of maternal age with adverse perinatal outcomes. Overall, 53/62 (85.5%) women in our study had cesarean delivery, which is similar to that reported by other investigators and may represent late referrals from peripheral hospitals in developing countries [8,9]. The proportion of preterm births was highest in group B (70.6%), with the indications being severe preeclampsia (four cases), absent end diastolic flow in umbilical artery (three cases), meconium-stained liquor (three cases), blood-stained liquor (one case), and reduced maternal perception of fetal movements (one case). Pregnancy was allowed to continue to term if both NST and CPR were normal (group A) and pregnancy was terminated if NST was abnormal before labor (groups C and D).

The mean birth weight was highest in group A (2720.3 g) and lowest in group D (1468 g). Overall, 39/62 (62.9%) neonates had low birth weight (LBW), with the highest LBW babies in group D (88.9%) and the lowest in group A (45.5%). This may represent the subset of babies with undiagnosed FGR in group D, for whom decision of termination of pregnancy based on CPR would have been ideal, rather than based on abnormal NST. Hypertension in pregnancy is associated with FGR in 30-40%, and in these cases, the use of CPR allows the assessment of blood flow abnormalities in the maternal-fetal-placental unit. CPR reflects early fetal adaptation to chronic hypoxia and detects clinically unrecognized fetal compromise. CPR has been shown to be a better predictor for adverse perinatal outcomes compared with UA or MCA Doppler alone [13]. These changes in fetal cerebral circulation occur at a time when the nonstress test is still normal [9]. In the temporal sequence of fetal Doppler abnormalities, abnormal CPR is the second earliest change to occur after abnormal MCA Doppler, while the second last change is abnormal NST [14]. It is this lead time that gives an opportunity to improve fetal outcomes. In our study also, the three neonatal deaths were in group D, but none in the other groups, indicating that in women with HDP, terminating the pregnancy before both CPR and NST have turned abnormal saves neonatal lives.

CPR had higher sensitivity (93.3% versus 46.67%), with greater specificity (74.47% versus 65.91%), PPV (53.85% versus 30.43%), and NPV (97.22% versus 79.49%) than NST in predicting NICU admission rates in women with HDP. Previous studies have reported higher rates of NICU admission in fetuses with abnormal CPR in high-risk pregnancies (20.0%) and in those with pregnancy-induced hypertension (45.5%) [15,16]. In our study, 11 out of the 15 neonates who were admitted to NICU developed complications during their course of stay like feeding difficulty in three, neonatal sepsis in two, neonatal jaundice and symptomatic hypoglycemia in two each, and asymptomatic polycythemia and respiratory distress syndrome in one each. CPR had higher sensitivity (84.62% versus 61.54%), greater specificity (91.34% versus 69.39%), PPV (42.31% versus 34.78%), and NPV (94.44% versus 87.18%) than NST in predicting neonatal complications rates. The sensitivity and NPV of CPR for predicting BMV and requirement for CPAP were 100%. This means that if the CPR was normal, there was no likelihood of the neonate requiring BMV and CPAP. Our study thus quantifies the diagnostic accuracy of CPR versus NST across the spectrum of HPD in predicting adverse perinatal outcomes.

Strengths of our study

Our study compared CPR with NST in predicting adverse perinatal outcomes in women with all sub-classifications of HDP. Previous studies have looked into either the CPR or the NST for predicting adverse perinatal outcomes or have looked into fetal Doppler versus NST in high-risk pregnancies or have considered a sub-classification of HDP [4,5,7-10,15-17]. While CPR has been earlier shown to be a marker of impaired fetal growth and adverse pregnancy outcomes, it was unclear to which subgroup of pregnant women this applied and what is the effectiveness of the CPR in guiding clinical management [18,19]. Our study is an attempt in this direction, especially in resource-poor regions where HDP is a major cause of perinatal morbidity and mortality, and NST remains the standard of care.

Limitations of our study

We have taken a single cut-off value of CPR (<1 versus >1), whereas many recent studies have used gestational age-specific cut-off values for greater diagnostic accuracy [20-22]. We have classified neonates as LBW only, and not as FGR due to nonavailability of reliable dating scan in many participants, and late referrals.

## Conclusions

To conclude, in this prospective observational study conducted on women with HDP, CPR was found to be a good predictor of adverse perinatal outcomes in comparison to NST, which is commonly used in developing regions. CPR had greater sensitivity, specificity, PPV, specificity and NPV than NST for predicting NICU admission in women with HDP. CPR also had higher sensitivity and specificity than NST in predicting neonatal complications. The NPV of CPR was 100% for predicting requirement of BMV and CPAP. Thus, CPR had greater diagnostic accuracy than NST in predicting adverse perinatal outcomes in HDP.
